# Association Between Carotid Atherosclerosis and Post‐Stroke Cognitive Impairment in Patients With Mild Ischemic Stroke: A Prospective Cohort Study

**DOI:** 10.1002/cns.70699

**Published:** 2026-01-08

**Authors:** GuanHua Nie, ZhiHong Wei, Zhan Su, Haining Zhang

**Affiliations:** ^1^ Department of Neurology The First Hospital of Jilin University Changchun China; ^2^ Department of Dermatology, Allergy, and Venereology University of Lübeck Lübeck Germany

**Keywords:** carotid plaque Crouse score, degree of common carotid artery stenosis, mild acute ischemic stroke, post‐stroke cognitive impairment

## Abstract

**Background:**

Post‐stroke cognitive impairment (PSCI) is a common yet frequently overlooked complication that adversely affects recovery and long‐term outcomes in stroke survivors. Early identification of individuals at risk is essential for timely cognitive rehabilitation.

**Objective:**

This study aimed to investigate the association between carotid atherosclerosis indicators—specifically, carotid plaque burden and degree of carotid artery stenosis—and the occurrence of PSCI in patients with mild acute ischemic stroke (AIS), using carotid ultrasound as a cost‐effective, widely accessible diagnostic modality.

**Methods:**

A prospective cohort of 181 patients diagnosed with AIS within 7 days of onset was enrolled. Baseline demographics, clinical characteristics, and carotid ultrasound parameters were collected. PSCI was assessed at 6 months using the Montreal Cognitive Assessment (MoCA). Binary logistic regression was used to identify independent predictors of PSCI. The predictive accuracy of individual and combined markers was evaluated using receiver operating characteristic (ROC) curve analysis.

**Results:**

Among 181 participants, 75 (41.4%) were diagnosed with PSCI at the 6‐month follow‐up. Multivariate analysis revealed that the carotid plaque Crouse score (OR = 1.157, 95% CI: 1.055–1.269) and severe carotid artery stenosis (OR = 3.733, 95% CI: 1.582–8.811) were independently associated with PSCI. ROC analysis demonstrated modest predictive performance for the Crouse score (AUC = 0.667) and stenosis (AUC = 0.596), while a multivariable model incorporating clinical and ultrasound parameters achieved an AUC of 0.818 (95% CI: 0.758–0.877). Significant between‐group differences were observed in AVLT‐I, AVLT‐II, VFT, TMT‐B, CDT, and MoCA subdomains (*p* < 0.05).

**Conclusion:**

Carotid plaque burden and severe carotid stenosis are independently associated with the development of PSCI in patients with mild AIS. Carotid ultrasound, combined with clinical risk factors, may provide a practical approach for early identification and risk stratification of PSCI.

**Trial Registration:** Chinese Clinical Trial Registry (ChiCTR1900022675); URL: https://www.chictr.org.cn/

## Graphical abstract

Carotid ultrasound parameters, particularly Crouse score and severe carotid stenosis, are independently associated with post‐stroke cognitive impairment and may serve as simple, non‐invasive predictors for early risk stratification.

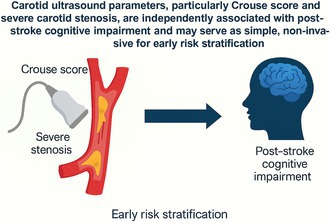

## 1 Introduction

Post‐stroke cognitive impairment (PSCI) is a syndrome characterized by a range of cognitive symptoms that emerge within 6 months after a stroke event and meet the diagnostic criteria for cognitive impairment [1]. It typically develops in the aftermath of the stroke. Despite its high prevalence, both patients and their families often prioritize physical and speech impairments during the acute or early recovery phase, while cognitive symptoms—such as memory deficits, inattention, and behavioral abnormalities—are frequently overlooked. This lack of awareness may result in missed opportunities for timely cognitive intervention. Therefore, early recognition and diagnosis of PSCI are essential in clinical practice.

The precise pathophysiological mechanisms underlying vascular cognitive impairment remain incompletely understood. However, accumulating evidence suggests that hypoperfusion, oxidative stress, and inflammation are key contributors to its development. These factors—acting independently or synergistically—may impair neurovascular unit function, ultimately triggering cognitive decline. Studies have demonstrated that inflammation and immune responses play critical roles in the pathogenesis of ischemic stroke [2, 3]. Even before clinical onset, dysregulated immune activity may initiate inflammatory processes in and around vascular walls, promoting thrombosis, altering vascular reactivity, and accelerating atherosclerotic progression. Over time, the gradual accumulation of carotid plaques, increased arterial stiffness, and luminal narrowing may reduce cerebral perfusion and disrupt neurovascular coupling, thereby exacerbating cognitive dysfunction [4].

Although PSCI is commonly associated with large‐artery pathology, the precise relationship between large‐vessel disease and PSCI remains unclear. While some studies suggest that arterial stenosis strongly predicts PSCI [5], others have reported conflicting results [6]. Clarifying the role of atherosclerotic features may enhance our understanding of PSCI pathogenesis and support early identification and prevention strategies.

Two primary approaches to predicting PSCI have been recommended in both domestic and international guidelines. One approach involves early cognitive screening using neuropsychological scales during the acute phase of stroke, while the other relies on multivariate prediction models based on known risk factors. However, full neuropsychological assessments are time‐consuming and may be difficult to implement in the acute setting, as patients are often uncooperative or emotionally unstable. Risk factor‐based models, on the other hand, frequently depend on advanced radiological parameters that are not always accessible in routine practice. Given China's large stroke population and limited medical resources, there is an urgent need for a low‐cost, easily obtainable marker to support early PSCI identification. This study aims to evaluate the diagnostic value of carotid atherosclerosis markers for PSCI to facilitate timely screening and intervention.

## 2 Materials and Methods

### 2.1 Study Design and Population

**FIGURE 1:**
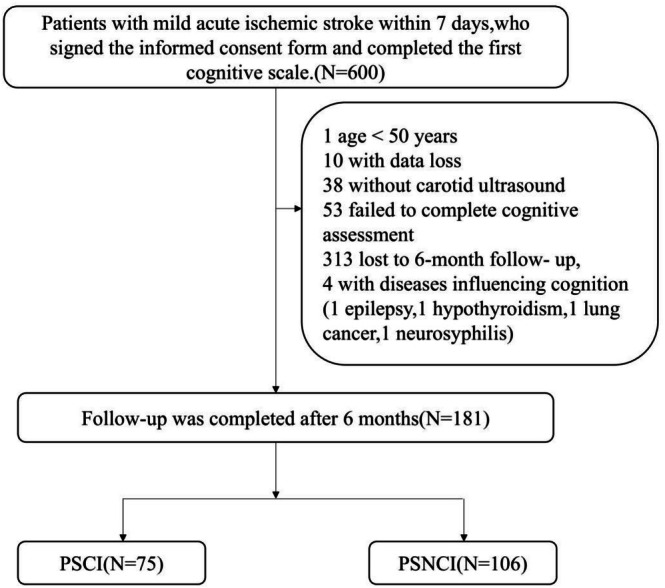
Study flow diagram. PSCI, post‐stroke cognitive impairment; PSNCI, post‐stroke no cognitive impairment.

In this study, 181 patients with mild acute ischemic stroke (AIS) were consecutively enrolled from October 2020 to October 2022 at the Department of Neurology, Bethune First Hospital of Jilin University. Mild AIS was defined as the presence of acute ischemic lesions on diffusion‐weighted MRI accompanied by relatively minor neurological deficits, operationalized as a NIHSS score ≤ 6 at admission. This definition is consistent with prior studies examining cognitive outcomes after mild stroke [7, 8]. All participants were assessed 6 months after stroke onset. Patients with a Montreal Cognitive Assessment (MoCA) score of less than 22 were classified as having post‐stroke cognitive impairment (PSCI), while those with a score of 22 or higher were categorized as having post‐stroke non‐cognitive impairment (PSNCI) [9]. All enrolled patients received conservative medical therapy in accordance with standard clinical practice, including antiplatelet agents and lipid‐lowering medications. Patients with complete occlusion were excluded to ensure that carotid plaque burden and stenosis could be reliably assessed via ultrasound. No participant underwent carotid endarterectomy or stenting during the follow‐up period. A detailed study flowchart is presented in Figure 1. The inclusion criteria were as follows: (i) diagnosis of AIS within 7 days of onset based on WHO criteria [10] and age between 50 and 80 years; (ii) presence of neurological deficits and acute ischemic lesions confirmed by diffusion‐weighted imaging (DWI) on MRI; (iii) National Institutes of Health Stroke Scale (NIHSS) score ≤ 6; (iv) ability to cooperate with cognitive assessments and provide written informed consent signed by both the patient and a legal guardian. The exclusion criteria were: (i) cognitive impairment secondary to other systemic, psychiatric, infectious, toxic, or metabolic conditions; (ii) a prior history of memory impairment or diagnosed cognitive disorder before stroke; use of medications known to affect cognition (e.g., anticholinesterase agents, sedatives, anxiolytics, antipsychotics) within the preceding 2 weeks; (iii) inability to complete cognitive assessments due to severe visual, speech, hearing, or physical disabilities; (iv) inability to undergo key study procedures, including laboratory tests or neuropsychological evaluations; (v) contraindications for MRI, such as pacemakers or metallic implants; (vi) complete occlusion of the common carotid artery. Patients with complete occlusion were excluded to ensure that carotid plaque burden and stenosis could be reliably assessed via ultrasound. Due to limited historical imaging data, it was not always possible to determine whether the occlusions were chronic or newly developed during the acute stroke event.

The study protocol was approved by the Ethics Committee of the First Hospital of Jilin University and conducted in accordance with the Declaration of Helsinki. Written informed consent was obtained from all participants.

### 2.2 Study Variables

General clinical data were collected during hospitalization, including age, sex, and educational level. The Trial of Org 10,172 in Acute Stroke Treatment (TOAST) classification was used to categorize ischemic stroke etiology into large artery atherosclerosis (LAA), cardioembolism (CE), small artery occlusion (SAA), other determined causes (OD), and undetermined causes (UD). Neurological deficit severity was assessed using the National Institutes of Health Stroke Scale (NIHSS) score [11], ranging from 0 to 6.

Cranial magnetic resonance imaging (MRI) was performed within 7 days of stroke onset. Imaging sequences included T1‐weighted, T2‐weighted, and diffusion‐weighted imaging (DWI). White matter hyperintensities (WMH) were evaluated according to the STRIVE neuroimaging criteria [12] and graded using the Fazekas scale. Total Fazekas scores (combined periventricular and deep WMH) were categorized as mild (0–2), moderate [3, 4], or severe [5, 6]. All MRI data were independently evaluated by experienced radiologists. All patients underwent diffusion‐weighted MRI to confirm acute ischemic lesions. Lesion location and volume were documented, but because only mild AIS patients (NIHSS ≤ 6) with small infarcts were included, these characteristics were not incorporated as covariates in the multivariate analysis. Baseline MoCA scores were used to account for both pre‐existing cognitive function and acute lesion effects.

Carotid ultrasonography was performed using a 7.5 MHz linear array transducer. The examination covered unilateral vascular structures, focusing on the common carotid artery, carotid bifurcation, internal carotid artery, and external carotid artery. The carotid intima‐media thickness (IMT) was measured at the far wall of the common carotid artery. Carotid plaque dimensions were recorded as length × thickness (mm). The Crouse score was calculated as the sum of the maximum plaque thicknesses from both carotid arteries.

The degree of carotid artery stenosis was classified according to expert consensus [13] as follows: mild (< 50%), moderate (50%–69%), and severe (70%–99%). Patients with complete carotid occlusion were excluded.

The following ultrasound parameters were documented in detail: left and right common carotid IMT values; total number of plaques in the bilateral carotid arteries; plaque location (none, unilateral, or bilateral); plaque dimensions (length, thickness, and area) at the carotid bifurcation; plaque distribution at the bifurcation (none, unilateral, or bilateral); Crouse score; and degree of carotid artery stenosis.

Between days 3 and 10 after stroke onset, after neurological symptoms had stabilized, a trained assessor conducted standardized cognitive evaluations in a quiet, distraction‐free setting. Global cognitive function was measured using the Montreal Cognitive Assessment (MoCA, maximum score 30), with a one‐point correction for participants with fewer than 12 years of formal education.

Post‐stroke cognitive impairment (PSCI) was defined according to the Expert Consensus on the Management of Post‐Stroke Cognitive Impairment, which recommends using MoCA 3–6 months post‐stroke and considers a score < 22 indicative of cognitive impairment. MoCA has been widely validated internationally, and a cutoff near 22 provides good sensitivity and specificity for clinically significant deficits, ensuring consistency with national and international standards. MoCA scores were recorded at baseline and at 6 months. Patients with MoCA < 22 at 6 months were classified as PSCI, while those with MoCA ≥ 22 were classified as PSNCI [9].

Specific cognitive domains were further assessed using the following tests: Auditory Verbal Learning Test‐I and II (AVLT‐I/II) for memory and learning; Verbal Fluency Test (VFT) for language function; Trail Making Test‐B (TMT‐B) for attention and executive function; Clock Drawing Test (CDT), corresponding to MoCA Item 1, for visuospatial ability. Subdomain scores from the MoCA were also documented, including visuospatial/executive, naming, attention, repetition/tapping, calculation, language, abstraction, delayed recall, and orientation [14]. In addition, changes in MoCA scores over the 6‐month period were analyzed for all participants.

### 2.3 Statistical Analysis

All statistical analyses were performed using SPSS version 26.0 (IBM Corp., Armonk, NY, USA) and R version 4.5.1. Continuous variables with a normal distribution (e.g., age) were expressed as mean ± standard deviation (SD) and compared between groups using independent‐samples *t*‐tests. Non‐normally distributed continuous variables (e.g., years of education, blood lipid levels, fasting blood glucose, cystatin C, uric acid, homocysteine, folic acid, magnesium, copper, ferritin, and Crouse score) were presented as median and interquartile range (IQR, 25th–75th percentile) and compared using the Mann–Whitney *U* test. Categorical variables (e.g., sex, medical history, TOAST classification, and degree of carotid stenosis) were reported as frequencies and percentages and compared using the chi‐square test.

To investigate the association between carotid atherosclerosis and PSCI, binary logistic regression models were constructed. To avoid potential multicollinearity between Crouse score and carotid stenosis, three logistic regression models were constructed. Model 1 adjusted for age, sex, and years of education. Model 2 further adjusted for hypertension, previous stroke, and white matter hyperintensities (WMH) based on Model 1. Variables with a *p*‐value < 0.1 in the univariate analysis, along with clinically relevant covariates, were included in the multivariate logistic regression. Covariates included in the multivariable logistic regression models were selected based on prior literature on post‐stroke cognitive impairment and clinical relevance, including age, sex, education, hypertension, prior stroke, and white matter hyperintensities. Carotid stenosis was categorized according to established clinical criteria as mild (< 50%), moderate (50%–69%), and severe (70%–99%). For multivariable analysis, mild and moderate stenosis were combined into a single “non‐severe” reference category based on prior literature and clinical relevance, while severe stenosis (70%–99%) was analyzed as a separate binary variable. Sensitivity analyses using inverse probability weighting and multiple imputation were conducted to account for potential bias due to missing follow‐up data.

The discriminative performance of models incorporating different carotid atherosclerotic features was evaluated using receiver operating characteristic (ROC) curve analysis. Statistical significance was set at a two‐tailed *p*‐value < 0.05.

To evaluate potential bias due to differential follow‐up or differential availability of standardized carotid ultrasound, we included the indicator “has_standard_ultrasound” in both IPW and MI models. The primary logistic regression models were refitted using the weighted or imputed datasets. Consistency of direction and significance across these analyses was considered evidence of robustness. Inverse‐probability weighting (IPW): The probability of completing follow‐up was modeled using baseline covariates, including age, sex, education, baseline ADL, baseline MoCA, hypertension, diabetes, prior stroke, smoking, and drinking. Predicted probabilities were used to generate weights for the analytic sample, and the primary logistic regression models were refitted using these weights. Multiple imputation (MI): Missing 6‐month MoCA scores were imputed under a missing‐at‐random assumption (20 imputations, predictive mean matching), and primary analyses were repeated.

## 3 Results

### 3.1 Baseline Characteristics of PSCI and PSNCI Groups

Table 1 summarizes the baseline demographic and clinical characteristics of the study participants. A total of 181 patients were included, with 75 in the PSCI group and 106 in the PSNCI group. There were no significant differences between groups in terms of age (63.4 ± 7.7 vs. 61.6 ± 7.5 years, *p* = 0.116), sex (male: 68.0% vs. 79.2%, *p* = 0.087), smoking history (45.3% vs. 52.8%, *p* = 0.320), or alcohol consumption (42.7% vs. 51.9%, *p* = 0.221).

**TABLE 1:** Baseline characteristics of PSCI and PSNCI groups.

Variable	PSCI (*n* = 75)	PSNCI (*n* = 106)	*p*
Age (years)	63.4 ± 7.7	61.6 ± 7.5	0.116
Male sex (%)	51 (68.0%)	84 (79.2%)	0.087
Smoking (%)	34 (45.3)	56 (52.8)	0.32
Drinking (%)	32 (42.7)	55 (51.9)	0.221
Hypertension (%)	54 (72.0%)	50 (47.2%)	**0.001**
Diabetes (%)	22 (29.3)	26 (24.5)	0.471
History of stroke (%)	30 (40.0%)	24 (23.1%)	**0.015**
Heart disease (%)	5 (6.7)	14 (13.2)	0.157
Years of education, median (IQR)	9 (6.0–12.0)	12 (9.0–15.0)	**0.001**
WMH severity (%)			**0.005**
Mild	18 (24.0%)	30 (28.3%)	
Moderate	21 (38.7%)	49 (46.2%)	
Severe	36 (34.8%)	27 (25.5%)	
TOAST (%)			0.327
Aortic atherosclerotic type	38 (50.7)	45 (42.5)	
Cardiogenic embolic type	5 (6.7)	14 (13.2)	
Arteriolar occlusion type	23 (30.7)	40 (37.7)	
Other causes	6 (8.0)	5 (4.7)	
Unexplained type	3 (4.0)	2 (1.9)	
MMSE, median (IQR)	24 (21.0–26.0)	28 (27.0–29.0)	**< 0.001**
Baseline MOCA, median (IQR)	17 (14.0–20.0)	24 (21.0–26.0)	**< 0.001**
CDR, median (IQR)	0.5 (0.5–1.0)	0.5 (0–0.5)	**< 0.001**
ADL, median (IQR)	22 (20.0–29.0)	20 (20.0–22.0)	**0.001**

*Note:* Bold values indicate statistically significant differences (*p* < 0.05).

Abbreviations: ADL, activities of daily living; CDR, clinical dementia rating; IQR, interquartile range; MMSE, mini‐mental state examination; PSCI, post‐stroke cognitive impairment; PSNCI, post‐stroke non‐cognitive impairment; WMH, white matter hyperintensity.

However, a significantly higher proportion of patients in the PSCI group had hypertension (72.0% vs. 47.2%, *p* = 0.001) and a history of stroke (40.0% vs. 23.1%, *p* = 0.015). The PSCI group also had fewer years of education [median: 9 (IQR: 6.0–12.0) vs. 12 (IQR: 9.0–15.0), *p* = 0.001]. White matter hyperintensity (WMH) burden differed significantly between groups (*p* = 0.005), with more patients in the PSCI group classified as having severe WMH (34.8% vs. 25.5%). As shown in Table 1, some PSNCI participants had moderate WMH severity. Mild to moderate WMH is common in older adults and may not result in detectable cognitive impairment within 6 months, indicating that WMH is one of multiple vascular risk factors contributing to PSCI rather than a sole determinant. Cognitive and functional assessments showed markedly worse performance in the PSCI group. The PSCI group had lower MMSE scores [24 (21.0–26.0) vs. 28 (27.0–29.0), *p* < 0.001], higher CDR scores [0.5 (0.5–1.0) vs. 0.5 (0–0.5), *p* < 0.001], and poorer ADL scores [22 (20.0–29.0) vs. 20 (20.0–22.0), *p* = 0.001]. Among the 106 patients classified as PSNCI at 6 months, 33 patients had baseline MoCA scores below 22 but improved to ≥ 22 at follow‐up, suggesting partial cognitive recovery.

Table S1 presents the full set of baseline demographic and clinical characteristics evaluated in all participants, including variables with and without statistically significant differences between the PSCI and PSNCI groups. Table 1 in the main text summarizes key variables that showed significant differences or have been previously identified as potential contributors to post‐stroke cognitive impairment. Inclusion of Table S1 provides a complete overview of all measured baseline parameters, enhancing transparency and reproducibility.

### 3.2 Carotid Atherosclerosis Markers in PSCI and PSNCI Groups

The comparison of carotid ultrasound parameters between the PSCI and PSNCI groups is presented in Table 2. The median carotid plaque Crouse score was significantly higher in the PSCI group than in the PSNCI group [6.0 (4.0–9.0) vs. 4.0 (2.0–6.0), *p* = 0.001]. Similarly, the right carotid intima‐media thickness (IMT) was greater in the PSCI group [0.96 (0.91–1.05) mm vs. 0.91 (0.85–0.99) mm, *p* = 0.006].

**TABLE 2:** Carotid atherosclerosis markers in PSCI and PSNCI groups.

Variable	PSCI (*n* = 75)	PSNCI (*n* = 106)	*p*
Carotid plaque Crouse score	6.0 (4.0–9.0)	4.0 (2.0–6.0)	**0.001**
Number of plaques	3 (2.0–5.0)	2 (1.0–3.0)	0.188
Left carotid IMT (mm)	1.1 (0.6–1.7)	0.7 (0.6–1.3)	0.197
Right carotid IMT (mm)	0.96 (0.91–1.05)	0.91 (0.85–0.99)	**0.006**
Plaque length at bifurcation (mm)	9 (6.7–12.6)	8.5 (6.1–11.5)	0.874
Plaque thickness at bifurcation (mm)	2.1 (1.6–2.6)	1.8 (1.7–2.7)	0.858
Plaque area at bifurcation (mm^2^)	20.0 (12.5–28.3)	15.8 (9.8–29.7)	0.978
Degree of carotid stenosis (%)			**0.002**
Mild (< 50%)	36 (48.0%)	73 (68.9%)	
Moderate (50%–69%)	10 (13.3%)	13 (12.3%)	
Severe (70%–99%)	29 (38.7%)	20 (18.9%)	

*Note:* Bold values indicate statistically significant differences (*p* < 0.05).

Abbreviations: IMT, intima‐media thickness; PSCI, post‐stroke cognitive impairment; PSNCI, post‐stroke non‐cognitive impairment.

There were no statistically significant differences between the two groups in the number of carotid plaques, left carotid IMT, or plaque morphology measures at the carotid bifurcation, including length, thickness, and area (all *p* > 0.05).

Regarding carotid artery stenosis, there was a significant difference in the degree of stenosis distribution between the two groups (*p* = 0.002). Severe carotid stenosis (70%–99%) was more prevalent in the PSCI group (38.7%) than in the PSNCI group (18.9%).

Table S2 provides a comprehensive summary of carotid and intracranial arterial ultrasound measurements in all participants. Carotid plaque morphology (length, thickness, area), plaque number and distribution, IMT, and degree of stenosis were systematically assessed. Blood flow velocity and pulsatility index were measured in bilateral anterior cerebral arteries (ACA), middle cerebral arteries (MCA), posterior cerebral arteries (PCA), as well as vertebral (VA) and basilar arteries (BA), representing the anterior, middle, and posterior circulations. While the main text (Table 2) presents variables with significant between‐group differences, Table S2 contains the complete dataset, including nonsignificant findings, ensuring transparency and reproducibility.

### 3.3 Association Between Carotid Plaque Characteristics and Cognitive Post‐Stroke Cognitive Impairment

Table 3 presents the results of crude and multivariate logistic regression analyses examining the association between carotid atherosclerosis indicators and the risk of PSCI. In the crude model, both the carotid plaque Crouse score (OR = 1.152, 95% CI: 1.068–1.241, *p* < 0.001) and severe carotid stenosis (OR = 3.033, 95% CI: 1.466–6.277, *p* = 0.003) were significantly associated with an increased risk of PSCI.

**TABLE 3:** Association between carotid plaque characteristics and post‐stroke cognitive impairment.

Variable	Crude		Model 1		Model 2	
	OR (95% CI)	*p*	OR (95% CI)	*p*	OR (95% CI)	*p*
Carotid plaque Crouse score	1.152 (1.068–1.241)	**< 0.001**	1.153 (1.063–1.251)	**0.001**	1.157 (1.055–1.269)	**0.002**
Number of plaques	1.084 (0.940–1.249)	0.266	1.065 (0.913–1.242)	0.421	1.057 (0.893–1.251)	0.521
Left carotid IMT (mm)	1.335 (0.907–1.964)	0.143	1.283 (0.843–1.953)	0.244	1.325 (0.842–2.117)	0.219
Right carotid IMT (mm)	1.367 (0.87–2.134)	0.168	1.269 (0.785–2.050)	0.331	1.380 (0.822–2.318)	0.223
Plaque length at bifurcation (mm)	0.988 (0.94–1.038)	0.630	0.971 (0.920–1.026)	0.301	0.962 (0.904–1.023)	0.217
Plaque thickness at bifurcation (mm)	0.996 (0.76–1.293)	0.976	0.861 (0.644–1.150)	0.311	0.861 (0.625–1.186)	0.359
Plaque area at bifurcation (mm^2^)	1.001 (0.987–1.015)	0.901	0.995 (0.980–1.011)	0.553	0.993 (0.976–1.010)	0.426
*Degree of stenosis of the common carotid artery (%)*						
Mild–moderate stenosis	Reference		Reference		Reference	
Severe stenosis	3.033 (1.466–6.277)	**0.003**	3.532 (1.635–7.632)	**0.001**	3.733 (1.582–8.811)	**0.003**

*Note:* Model 1: Adjusted for age, sex, and years of education. Model 2: Further adjusted for hypertension, prior stroke, and white matter hyperintensity (WMH) on the basis of Model 1. *p* < 0.05 is considered statistically significant. Bold values indicate statistically significant differences (*p* < 0.05).

Abbreviations: CI, confidence interval; IMT, intima‐media thickness; OR, odds ratio; PSCI, post‐stroke cognitive impairment.

After adjusting for age, sex, and years of education (Model 1), the Crouse score (OR = 1.153, 95% CI: 1.063–1.251, *p* = 0.001) and severe stenosis (OR = 3.532, 95% CI: 1.635–7.632, *p* = 0.001) remained independent predictors. These associations persisted in Model 2, which further adjusted for hypertension, prior stroke, and white matter hyperintensity. In this fully adjusted model, the Crouse score (OR = 1.157, 95% CI: 1.055–1.269, *p* = 0.002) and severe stenosis (OR = 3.733, 95% CI: 1.582–8.811, *p* = 0.003) continued to be significantly associated with PSCI.

Other variables, including plaque number, IMT, and plaque morphology at the carotid bifurcation (length, thickness, and area), were not significantly associated with PSCI in any of the models (all *p* > 0.05).

### 3.4 Subgroup Analysis of Crouse Score and Severe Carotid Stenosis

**FIGURE 2:**
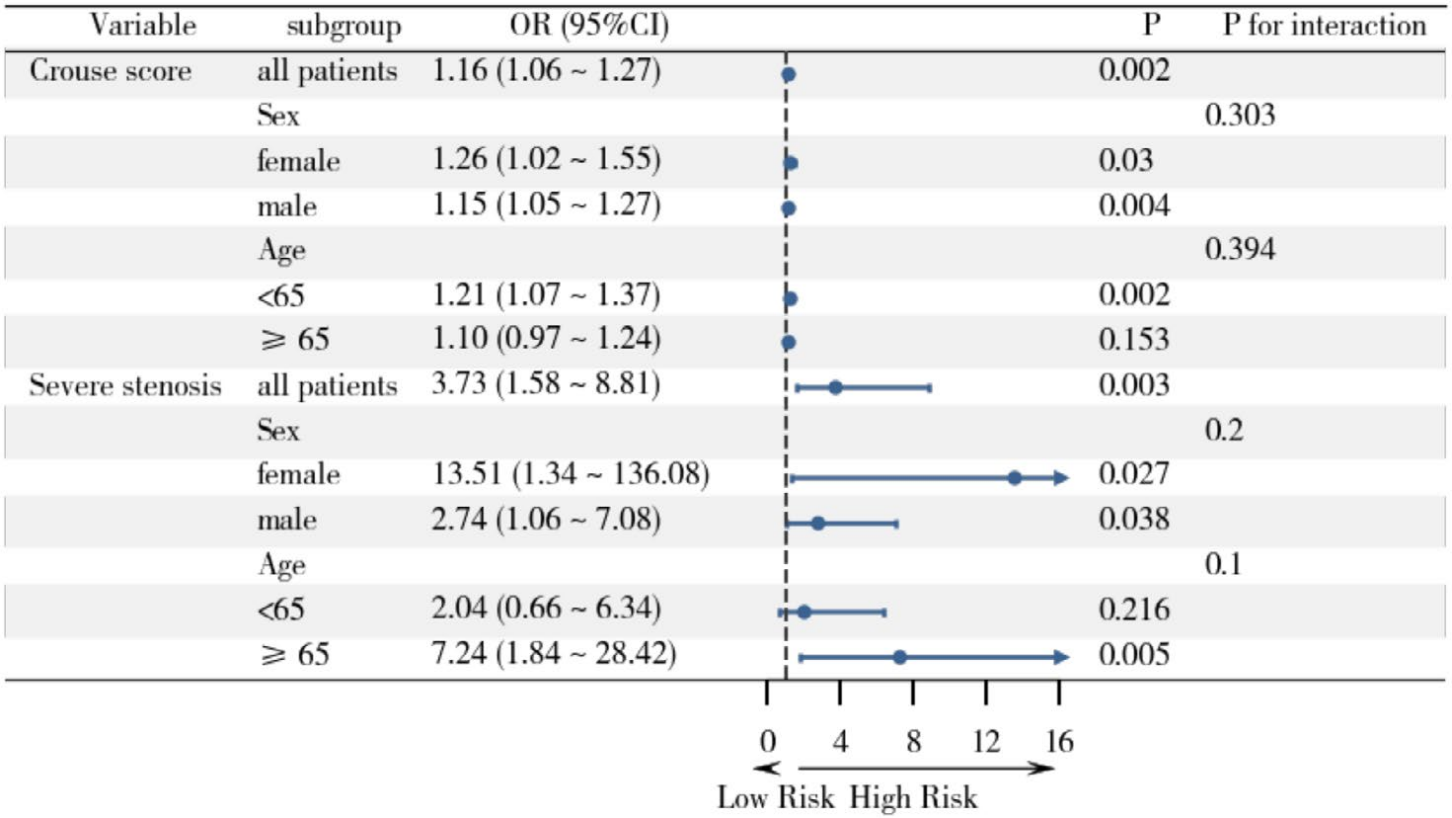
Subgroup analysis of Crouse score and severe carotid stenosis.

Subgroup analyses stratified by sex and age are presented in Figure 2. The association between carotid plaque Crouse score and PSCI remained significant across most subgroups. Among all patients, the Crouse score was independently associated with PSCI (OR = 1.16, 95% CI: 1.06–1.27, *p* = 0.002). The effect was significant in both males (OR = 1.15, 95% CI: 1.05–1.27, *p* = 0.004) and females (OR = 1.26, 95% CI: 1.02–1.55, *p* = 0.030), with no significant interaction by sex (P for interaction = 0.303). Similarly, the effect was observed in participants < 65 years (OR = 1.21, 95% CI: 1.07–1.37, *p* = 0.002), but not in those ≥ 65 years (OR = 1.10, 95% CI: 0.97–1.24, *p* = 0.153); however, the interaction by age was not statistically significant (*P* for interaction = 0.394).

For severe carotid stenosis, the association with PSCI remained significant in the overall cohort (OR = 3.73, 95% CI: 1.58–8.81, *p* = 0.003). Notably, the effect appeared stronger in females (OR = 13.51, 95% CI: 1.34–136.08, *p* = 0.027) than in males (OR = 2.74, 95% CI: 1.06–7.08, *p* = 0.038), although no significant interaction by sex was detected (*P* for interaction = 0.200). When stratified by age, the effect was more pronounced in patients aged ≥ 65 years (OR = 7.24, 95% CI: 1.84–28.42, *p* = 0.005) but not in those < 65 years (OR = 2.04, 95% CI: 0.66–6.34, *p* = 0.216). The age‐by‐stenosis interaction did not reach statistical significance (*P* for interaction = 0.100).

### 3.5 The Combination of Multiple Indicators in the Model Proves to Be Valuable in Predicting the ROC Curve of PSCI

**FIGURE 3:**
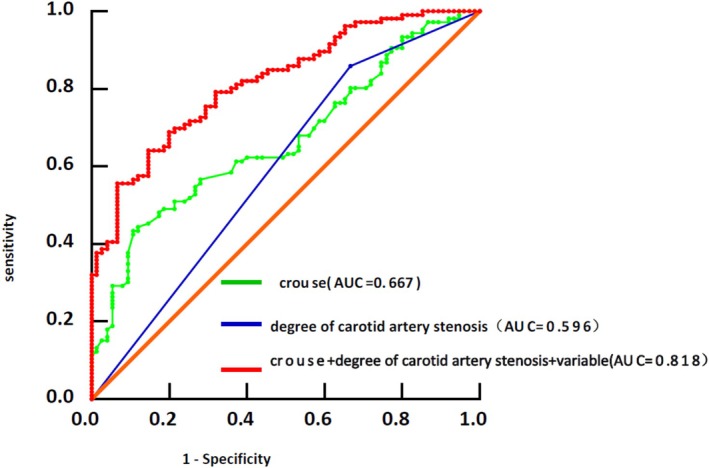
A model combining multiple indicators is used to predict the ROC curve of PSCI. Baseline variable + Crouse score (green), baseline variable + severe stenosis (blue), and baseline variable + Crouse score + severe stenosis (red).

ROC curve analysis revealed that the sensitivity and specificity of individual carotid atherosclerosis indices for predicting post‐stroke cognitive impairment (PSCI) were limited. Six independent predictors of PSCI were identified: years of education, white matter hyperintensities (WMH), degree of carotid artery stenosis, carotid plaque Crouse score, history of previous stroke, and history of hypertension. Based on these factors, three predictive models were developed: one model combining the carotid plaque Crouse score with baseline covariates, another combining common carotid artery stenosis with baseline covariates, and a combined model incorporating the carotid plaque Crouse score, common carotid artery stenosis, and baseline covariates including age, years of education, white matter hyperintensities (WMH), history of hypertension, and history of previous stroke. The area under the ROC curve (AUC) for the carotid plaque Crouse score model was 0.667 (95% CI: 0.589–0.745, *p* < 0.001), with a sensitivity of 43.4% and specificity of 89.3%. The common carotid artery stenosis model demonstrated an AUC of 0.596 (95% CI: 0.589–0.750, *p* = 0.001), with a sensitivity of 33.3% and specificity of 85.8%. In contrast, the combined model yielded a significantly higher AUC of 0.818 (95% CI: 0.758–0.877, *p* < 0.001), with sensitivity and specificity of 85.3% and 64.2%, respectively, at a cutoff value of 0.320. These findings indicate that the combined model integrating multiple indicators markedly improves predictive accuracy and sensitivity for PSCI compared to single‐marker models (Figure 3).

### 3.6 Neuropsychological Assessment Scale Between PSCI Group and PSNCI Group

Table 4 presents the Patient Neuropsychological Assessment Scale scores. At 6 months post‐stroke, PSCI patients had significantly lower AVLT‐I, AVLT‐II, VFT, and CDT scores and higher TMT‐B scores compared to PSNCI patients (all *p* < 0.001).

**TABLE 4:** Neuropsychological assessment scale between PSCI group and PSNCI group.

	All patients (*N* = 181)			PSCI (*N* = 75)			PSNCI (*N* = 106)			*p* ^*#*^
	Baseline	6 month	*p*	Baseline	6 month	*p*	Baseline	6 month	*p*	
AVLT‐II	4.76 ± 2.57	5.57 ± 2.70	< 0.001	3.58 ± 2.45	4.10 ± 2.71	< 0.001	5.51 ± 2.37	6.51 ± 2.25	< 0.001	< 0.001
AVLT‐I	13.65 ± 8.29	16.43 ± 8.26	< 0.001	9.58 ± 7.49	12.06 ± 8.05	< 0.001	16.23 ± 7.75	19.20 ± 7.15	< 0.001	< 0.001
VFT	39.37 ± 12.49	41.84 ± 11.64	< 0.001	31.82 ± 10.70	34.70 ± 8.43	< 0.001	44.15 ± 11.15	46.37 ± 11.15	< 0.001	< 0.001
TMT‐B	211.88 ± 82.50	195.06 ± 62.08	< 0.001	252.26 ± 74.51	232.33 ± 61.51	0.628	186.77 ± 73.99	171.89 ± 50.30	< 0.001	< 0.001
CDT	2.93 ± 1.48	3.22 ± 1.23	< 0.001	2.19 ± 1.37	2.36 ± 0.95	0.426	3.45 ± 1.32	3.82 ± 1.04	0.001	< 0.001

*Note:* The *p* ^#^ value represents the comparison between the PSCI group and the PSNCI group at 6 months, and the *p* value represents the comparison between baseline and 6 months. *p* < 0.05 indicates that the difference is statistically significant.

Abbreviations: AVLT‐I, Auditory verbal learning test‐I; AVLT‐II, Auditory verbal learning test‐II; CDT, Clock Drawing Test; TMT‐B, Trail making test‐B; VFT, Verbal fluency tests.

Within the PSCI group, AVLT‐I, AVLT‐II, and VFT scores improved significantly from baseline to 6 months (*p* < 0.001), while TMT‐B and CDT scores remained unchanged. The PSNCI group showed significant improvements in AVLT‐I, AVLT‐II, VFT, TMT‐B, and CDT over the same period (*p* < 0.001).

Overall, PSNCI patients outperformed PSCI patients across all neuropsychological tests at 6 months (*p* < 0.001).

### 3.7 Different Subdomains of MoCA in the PSCI Group and PSNCI Group

Table 5 summarizes MoCA subdomain scores reflecting cognitive performance. At 6 months post‐stroke, significant differences were observed between PSCI and PSNCI groups across visual–spatial ability, naming, attention, language, abstraction, delayed recall, and orientation (all *p* < 0.05), with PSCI patients scoring lower (*p* < 0.01).

**TABLE 5:** Different subdomain tables of MOCA in the PSCI group and PSNCI group.

Subdomains	Score range	All patients (*N* = 181)			PSCI (*N* = 75)			PSNCI (*N* = 106)				p*
		Baseline	6 month	*p*	Baseline	6 month	*p*	Baseline	6 month	*p*	*p* ^#^	
Visual space	0–5	2.93 ± 1.48	3.22 ± 1.23	< 0.001	2.19 ± 1.37	2.36 ± 0.95	0.426	3.45 ± 1.32	3.82 ± 1.04	0.001	< 0.001	< 0.001
Named	0–3	2.61 ± 0.71	2.57 ± 0.67	< 0.001	2.38 ± 0.90	2.28 ± 0.84	< 0.001	2.76 ± 0.49	2.76 ± 0.43	< 0.001	< 0.001	< 0.001
Attention	0–2	1.84 ± 0.38	1.87 ± 0.36	0.001	1.68 ± 0.50	1.74 ± 0.47	0.055	1.95 ± 0.21	1.95 ± 0.21	0.614	0.001	< 0.001
Read knock	0–1	0.71 ± 0.46	0.82 ± 0.39	0.002	0.57 ± 0.50	0.72 ± 0.45	0.042	0.81 ± 0.39	0.89 ± 0.32	0.838	0.006	< 0.001
Calculation	0–3	2.18 ± 1.04	2.33 ± 0.85	< 0.001	1.64 ± 1.05	1.85 ± 0.92	0.003	2.56 ± 0.84	2.67 ± 0.60	< 0.001	< 0.001	< 0.001
Language	0–3	2.28 ± 0.88	2.12 ± 0.94	< 0.001	1.88 ± 0.99	1.59 ± 0.98	< 0.001	2.57 ± 0.66	2.49 ± 0.71	< 0.001	< 0.001	< 0.001
Abstract	0–2	1.10 ± 0.76	0.98 ± 0.74	< 0.001	0.70 ± 0.74	0.57 ± 0.64	0.008	1.38 ± 0.65	1.27 ± 0.66	0.011	< 0.001	< 0.001
Delayed recall	0–5	1.55 ± 1.53	2.13 ± 1.62	< 0.001	0.78 ± 1.10	0.85 ± 1.19	0.159	2.08 ± 1.56	3.01 ± 1.25	< 0.001	< 0.001	< 0.001
Orientation	0–6	5.23 ± 1.20	5.44 ± 1.11	< 0.001	4.68 ± 1.43	4.92 ± 1.34	0.014	5.61 ± 0.80	5.81 ± 0.81	0.964	< 0.001	< 0.001

*Note:* *p* ^#^ represents the comparison between the PSCI group and the PSNCI group at 6 months. The p* value represents the comparison between the PSCI group and the PSNCI group at baseline, while *p* represents the comparison between baseline and at 6 months. *p* < 0.05 indicates that the difference is statistically significant.

In the PSCI group, orientation (4.68 ± 1.43 vs. 4.92 ± 1.34, *p* = 0.014) and calculation (1.64 ± 1.05 vs. 1.85 ± 0.92, *p* = 0.003) improved from baseline to 6 months, while naming, abstraction, and language declined; visuospatial ability, attention, and delayed recall remained stable.

PSNCI patients showed improvements in visual–spatial ability (3.45 ± 1.32 vs. 3.82 ± 1.04, *p* = 0.001), calculation (2.56 ± 0.84 vs. 2.67 ± 0.60, *p* < 0.001), and delayed recall (2.08 ± 1.56 vs. 3.01 ± 1.25, *p* < 0.001). However, naming, language, abstraction, and attention declined, while reading, knocking, and orientation were stable.

Overall, PSNCI patients outperformed PSCI patients across all seven MoCA subdomains at 6 months (*p* < 0.05). These findings go beyond simple group‐level comparisons, demonstrating that PSCI is characterized by impairments across multiple cognitive domains.

### 3.8 Sensitivity Analysis

Baseline characteristics between participants who completed the 6‐month assessment and those who did not were compared, as described in Table S3. After applying inverse probability weighting to adjust for potential follow‐up bias, a weighted logistic regression model was constructed to explore factors associated with PSCI. As shown in Table S4, higher Crouse scores were independently associated with an increased risk of PSCI (OR = 1.14, *p* = 0.01). Baseline MoCA score and hypertension also demonstrated significant associations with PSCI, while vascular risk factors such as age, diabetes, prior stroke, smoking, and drinking were not statistically significant after adjustment (*p* > 0.05). The IPW‐weighted analysis and MI support the robustness of this relationship, suggesting that carotid atherosclerosis could serve as a potential imaging biomarker for early identification of patients at risk for post‐stroke cognitive decline (see Figures S1 and S2).

These findings indicate that carotid atherosclerosis burden, quantified by the Crouse score, is a strong predictor of cognitive decline following stroke, independent of other vascular comorbidities.

## 4 Discussion

### 4.1 Correlation of Carotid Atherosclerosis and Cognitive Impairment

This study evaluated the association between carotid atherosclerosis and post‐stroke cognitive impairment (PSCI) in patients with acute ischemic stroke. We found that two carotid ultrasound parameters—the carotid plaque Crouse score and the presence of severe carotid stenosis—were independently associated with PSCI. Importantly, these associations remained significant even after adjusting for baseline cognitive function (MoCA), white matter hyperintensity (WMH) burden, educational level, age, sex, and vascular risk factors, indicating that carotid atherosclerosis contributes to post‐stroke cognitive outcomes independently of pre‐stroke cognitive status and chronic small vessel disease. This suggests that carotid atherosclerosis may play a direct role in cognitive decline after stroke, rather than merely reflecting pre‐existing brain vulnerability.

Compared with previous studies that rarely accounted for both baseline cognitive function and WMH burden, our findings provide stronger evidence for the independent contribution of carotid atherosclerosis to PSCI. These findings have important clinical implications for the early identification and management of high‐risk patients and suggest that interventions targeting carotid atherosclerosis may help prevent or mitigate post‐stroke cognitive impairment. Furthermore, the use of simple, non‐invasive carotid ultrasound may serve as a practical tool for identifying patients at elevated risk of cognitive decline following stroke, facilitating timely preventive or therapeutic interventions.

Carotid atherosclerosis progresses through several stages, from intima‐media thickening to plaque formation and arterial stenosis. Among these, carotid intima‐media thickness (IMT) has been widely studied as an early ultrasound marker of subclinical atherosclerosis. Several studies have reported associations between increased IMT and cognitive impairment. A 2016 cross‐sectional study in China found a strong correlation between IMT and global cognitive function in elderly individuals with lower educational levels [15]. Similarly, the INVADE project demonstrated that baseline IMT predicted cognitive decline at 6 years, independent of vascular risk factors, in initially cognitively normal individuals [16]. In a longitudinal cohort in the United States, greater IMT was linked to faster decline across multiple cognitive domains over an eight‐year follow‐up [17].

Although prior studies support the association between IMT and cognitive decline, our findings did not identify IMT as an independent risk factor for PSCI. This discrepancy may relate to sample size limitations, follow‐up duration, or population characteristics. Notably, our analysis included multiple ultrasound parameters, but only the Crouse score and severe carotid stenosis remained significant after adjustment. These findings reinforce the importance of focused vascular assessment and highlight the Crouse score as a potentially practical index for routine use.

Given that carotid ultrasound is non‐invasive, inexpensive, and widely available, it may serve as a convenient tool for cognitive risk screening in stroke patients. Early identification of individuals at risk of PSCI could facilitate timely intervention and improve long‐term neurological outcomes.

Carotid ultrasound enables direct visualization and quantification of atherosclerotic plaques, which result from the accumulation of lipids and inflammatory cells during arterial wall remodeling. Several studies have reported associations between plaque burden and cognitive function. In the Dallas Heart Study, an eight‐year follow‐up using the Montreal Cognitive Assessment (MoCA) revealed a linear relationship between the number of carotid plaques and declining cognitive scores [18]. A multicenter study in China reported that cognitive impairment was more prevalent in patients with ≥ 2 plaques and those with hypoechoic plaques, suggesting a role for both burden and plaque composition [19]. Similarly, a cohort of 6025 individuals followed through in‐person and telephone assessments found that the presence of carotid plaque independently predicted dementia over a seven‐year period [20]. In another study involving 4274 asymptomatic participants, both the number and total area of carotid plaques were associated with cognitive decline over 7 years [21].

However, not all findings have been consistent. The Rotterdam study, with an average follow‐up of 9 years, did not detect a significant association between plaque number and cognitive impairment [22]. These discrepancies may be attributed to differences in population characteristics, follow‐up duration, or plaque measurement methods.

In our study, although plaque number, location, and morphology were comprehensively recorded, only the carotid plaque Crouse score—reflecting the total plaque thickness—was independently associated with PSCI. This finding suggests that the cumulative burden of plaque, rather than count alone, may better reflect the vascular contribution to cognitive decline in stroke survivors.

Our findings revealed that the carotid plaque Crouse score was significantly higher in patients with PSCI compared to those without cognitive impairment. The Crouse score, which quantifies the total thickness of carotid plaques via Doppler ultrasound, serves as a sensitive and non‐invasive indicator of atherosclerotic burden. A meta‐analysis involving 5856 patients with mild cognitive impairment and 7001 controls found a significant association between increased carotid intima‐media thickness and cognitive decline [23]. While most prior studies have focused on IMT or plaque presence, evidence linking plaque burden—specifically the Crouse score—to cognitive outcomes remains limited.

Our study contributes to this area by demonstrating that elevated Crouse scores are independently associated with PSCI. Given its simplicity, objectivity, and accessibility, the Crouse score holds promise as a practical tool for early cognitive risk assessment in stroke survivors.

Interestingly, right carotid IMT was significantly higher in patients with PSCI. This lateralization may relate to anatomical and hemodynamic factors, as the right common carotid artery arises from the brachiocephalic trunk and experiences distinct flow dynamics. Previous studies have shown asymmetric associations between carotid blood flow and cognitive status [24] and highlighted the importance of right‐hemisphere functional integrity in post‐stroke cognition [25]. Although handedness can influence IMT asymmetry [26], our predominantly right‐handed cohort likely minimized this effect. Nevertheless, handedness should be recorded in future studies to better interpret the potential lateralization of carotid pathology.

As carotid atherosclerotic plaques increase in size and number, hemodynamics undergo alterations, which can result in carotid artery stenosis. Additionally, research literature has indicated a close association between different degrees of carotid artery stenosis and cognitive impairment. In a cross‐sectional cohort study conducted by Johnston across four communities in the United States, asymptomatic severe stenosis of the left carotid artery was found to be linked to cognitive impairment following regression analysis [27]. In alignment with the aforementioned study, the findings of this research suggest that individuals with severe carotid artery stenosis are at an increased risk of developing post‐stroke cognitive impairment. Severe carotid artery stenosis can be viewed as an indicator of accelerated cognitive decline in patients with dementia. Evaluating cerebral hemodynamics can aid in identifying individuals at a heightened risk of rapid cognitive deterioration who may benefit from screening for revascularization therapy. In a study involving Framingham's descendants, researchers observed a higher likelihood of executive dysfunction among participants with severe internal carotid artery stenosis [28]. Rocque found that visual–spatial impairments occur earliest in cognitive impairments caused by carotid artery stenosis [29]. Cheng investigated bilateral brain connectivity using magnetic resonance imaging and discovered that asymptomatic carotid artery stenosis (> 70%) not only diminishes interhemispheric connectivity but also precipitates cognitive decline [30]. Our investigation corroborates these findings, revealing a robust correlation between cognitive impairment and the severity of carotid artery stenosis. Moreover, severe carotid artery stenosis confers a heightened risk of cognitive impairment compared to mild to moderate stenosis. Exploring the relationship between cognitive impairment and intracranial and extracranial atherosclerosis underscores the significance of managing controllable vascular factors in the early recognition and intervention of cognitive decline. For individuals with carotid atherosclerosis progressing to carotid artery stenosis, further research is imperative to ascertain whether treatment modalities can ameliorate cognitive impairment. While carotid atherosclerosis and severe stenosis were independently associated with PSCI, we recognize that underlying neurodegenerative processes may also play a role, particularly in patients with subtle preclinical pathology. Our findings primarily reflect vascular contributions, and integration of neurodegenerative markers in future research will provide a more comprehensive understanding. The Crouse score reflects overall plaque burden, whereas stenosis reflects focal hemodynamic compromise. By constructing separate regression models and a combined model, we minimized potential multicollinearity and demonstrated that each marker provides distinct and complementary information. The combined model achieved superior predictive performance, supporting the additive prognostic value of structural and hemodynamic indicators of carotid disease.

Mechanistically, chronic plaque accumulation may promote systemic endothelial dysfunction and embolic risk, whereas severe stenosis may induce local hypoperfusion and watershed ischemia, both contributing to post‐stroke cognitive decline. These findings are consistent with prior studies suggesting that global atherosclerosis and focal stenosis exert synergistic effects on cerebral microcirculation and cognition.

### 4.2 Risk Factors for PSCI

Previous studies have shown that patients with mild to moderate stroke and pre‐existing white matter lesions are at increased risk of post‐stroke cognitive impairment (PSCI), regardless of new ischemic injury [31]. Similarly, the SMART‐MR study reported that the combination of cerebral atrophy with white matter hyperintensities (WMH) or infarctions can accelerate cognitive decline [32]. These findings are consistent with our results, supporting WMH as an independent risk factor for PSCI. It should be noted that periventricular WMH (PWMH) and deep WMH (DWMH) may differentially affect cognitive domains due to distinct pathophysiological mechanisms (e.g., PWMH related to perivascular and venous changes, DWMH reflecting microvascular pathology). In this study, we combined PWMH and DWMH into a total Fazekas score to simplify analysis and maintain statistical power. Future studies with larger cohorts could examine PWMH and DWMH separately to investigate their specific contributions to PSCI [33, 34].

Educational attainment is considered a key component of cognitive reserve, which may buffer against neurodegenerative changes [35]. Individuals with lower education levels have been found to have a 3.03‐fold increased risk of PSCI compared to those with higher education [36]. Our findings reinforce the role of education as an independent protective factor, with lower educational levels significantly associated with higher PSCI risk, in agreement with previous research.

Hypertension is another established independent risk factor for cognitive impairment after acute ischemic stroke (AIS) [37]. Elevated systolic blood pressure may impair brain reserve by negatively affecting hippocampal and dentate gyrus function [38, 39], with effects potentially beginning in early adulthood and contributing to hippocampal volume loss later in life [40]. Hypertension may also disrupt neural connectivity in the temporal lobe, thalamus, prefrontal cortex, and especially the hippocampus [41]. Animal models have demonstrated increased amyloid deposition in the hippocampus and related vascular structures under hypertensive conditions [42]. In non‐demented individuals carrying the ApoE ε4 allele, elevated blood pressure correlates with reduced hippocampal volume and impaired cognitive processing [43]. These findings align with our results, further confirming pre‐existing hypertension as an independent predictor of PSCI.

This study identified prior stroke as an independent risk factor for PSCI, consistent with previous findings. A 2022 meta‐analysis involving patients with prior stroke reported a more rapid cognitive decline [44]. Drawing on individual participant data from the Stroke and Cognition Alliance, the study analyzed nine longitudinal hospital cohorts across seven countries, with a median follow‐up of 2.68 years. Results showed that individuals with a history of stroke experienced significantly faster cognitive deterioration within 1–3 years post‐stroke compared to controls without prior stroke. This decline was further associated with advancing age and recurrent stroke.

### 4.3 Performance on Different Cognitive Scales of PSCI

PSCI is characterized by impairment in at least one cognitive domain. Deficits in processing speed and attention are common in the acute post‐stroke phase, with partial recovery typically observed by 3 months [45]. However, longitudinal studies have reported a persistent decline in visuospatial ability, attention, and processing speed up to 24 months post‐stroke, suggesting a long‐term deterioration. Recurrent strokes are associated with more severe declines in memory, attention/processing speed, executive function, and orientation [46]. Notably, even patients with “excellent” clinical recovery at 3 months may show deficits in memory, visuospatial skills, executive function, and language at 15 months [47, 48].

In our study, patients with PSCI performed significantly worse across all cognitive domains than those in the PSNCI group at 6 months. While PSCI patients showed improvements in AVLT‐I, AVLT‐II, and VFT, their TMT‐B and CDT scores remained unchanged. In contrast, the PSNCI group demonstrated significant improvements across all five tests (AVLT‐I, AVLT‐II, VFT, TMT‐B, and CDT). These findings suggest that cognitive recovery varies across domains and may be influenced by both stroke severity and timing of assessment.

In this study, MoCA subdomain scores were significantly higher in the PSNCI group compared to PSCI patients at the 6‐month follow‐up. We also examined longitudinal changes in MoCA subdomains from baseline to 6 months. However, to date, few studies have systematically explored how individual MoCA domains contribute to the detection of cognitive impairment over different follow‐up periods. Our findings may help clinicians better interpret domain‐specific cognitive changes and enhance the utility of MoCA in post‐stroke cognitive assessments.

While we used a MoCA score < 22 to define PSCI in line with national guidelines, we recognize that this cutoff may not capture subtle impairments in specific cognitive domains. To partially address this, we analyzed MoCA subdomain scores and baseline ADL. Future studies should consider integrating domain‐specific assessments and functional evaluations to provide a more comprehensive characterization of post‐stroke cognitive impairment.

This study has several limitations. First, it was a single‐center study with a relatively small sample size, which may limit generalizability; multicenter studies with larger cohorts are warranted. Second, patients with severe language or physical impairments were excluded, potentially introducing selection bias and underrepresenting more severe cases. Third, lesion location and volume were not directly included in multivariate models; however, the mild stroke severity and inclusion of baseline cognitive function likely mitigated major confounding. Fourth, neurodegenerative biomarkers were not assessed, limiting differentiation between vascular‐ and neurodegeneration‐related cognitive impairment. Fifth, a substantial proportion of participants did not complete the 6‐month cognitive assessment due to pandemic restrictions, lengthy testing, or medical/mobility limitations. Analyses of carotid atherosclerosis were restricted to participants with standardized in‐house ultrasound, which may introduce selection bias despite IPW, MI, and sensitivity analyses. Finally, cognitive function was assessed only at a single 6‐month follow‐up, limiting evaluation of long‐term trajectories. Future multicenter studies with repeated assessments, standardized imaging, and integration of vascular and neurodegenerative markers are needed to validate and extend these findings.

## 5 Conclusion

In conclusion, severe carotid artery stenosis and elevated carotid plaque Crouse scores during the acute phase of acute ischemic stroke (AIS) were independently associated with an increased risk of post‐stroke cognitive impairment (PSCI). These findings highlight the potential diagnostic value of carotid atherosclerosis markers in predicting PSCI and underscore their relevance for early risk stratification and clinical intervention.

## Author Contributions

G.N.: concept and design (lead), methodology (lead), statistical analysis (lead), drafting of the manuscript (lead). Z.W. and Z.S.: drafting of the manuscript, statistical analysis. H.Z.: concept and design, critical revision of the manuscript for important intellectual content, and study supervision. All authors read and approved the final manuscript.

## Funding

This work was supported by grant J2025JKJ017 (“Molecular mechanisms and strategies of LRP1 in regulating Aβ clearance impairment in Alzheimer’s disease brain deposition study”).

## Ethics Statement

The research was conducted in accordance with the World Medical Association Declaration of Helsinki and was approved by the First Hospital of Jilin University Ethics Committee (protocol code 19K023‐003). The procedures followed conformed to national and institutional guidelines, and written informed consent was obtained from all participants. The study was registered with the Chinese Clinical Trial Registry (URL: https://www.chictr.org.cn/; unique identifier: ChiCTR1900022675). The registration date for the clinical trial with the Clinical Trial Number ChiCTR1900022675 is 21st April 2019. The Clinical Trial Registry is the First Hospital of Jilin University.

## Conflicts of Interest

The authors declare no conflicts of interest.

## Supporting information


**Table S1:** General data were compared between PSCI and PSNCI groups.
**Table S2:** Comparison of atherosclerosis characteristics between PSCI and PSNCI groups.
**Table S3:** General data were compared between follow‐up and nonfollow‐ups.
**Table S4:**. IPW‐weighted logistic regression with multiple imputation for predictors of PSCI at 6 months.
**Figure S1:** Distribution of Crouse scores by post‐stroke cognitive impairment status.
**Figure S2:** Proportion of post‐stroke cognitive impairment according to carotid stenosis severity.

## Data Availability

The data that support the findings of this study are not openly available due to reasons of sensitivity and are available from the corresponding author upon reasonable request.
