# Supplementary material for: Association Between Carotid Atherosclerosis and Post‐Stroke Cognitive Impairment in Patients With Mild Ischemic Stroke: A Prospective Cohort Study
Source: CNS Neurosci Ther. 2026 Jan 8;32(1):e70699. doi: 10.1002/cns.70699 (PMC12780957; doi:10.1002/cns.70699)
Supplement: Supplementary file 1 — Table S1: General data were compared between PSCI and PSNCI groups. Table S2: Comparison of atherosclerosis characteristics between PSCI and PSNCI groups. Table S3: General data were compared between follow‐up and nonfollow‐ups. Table S4:. IPW‐weighted logistic regression with multiple imputation for predictors of PSCI at 6 months. Figure S1: Distribution of Crouse scores by post‐stroke cognitive impairment status. Figure S2: Proportion of post‐stroke cognitive impairment according to carotid stenosis severity. [file CNS-32-e70699-s001.docx]

**Table S1. General data were compared between PSCI and PSNCI groups**

|  | PSCI | PSNCI | ${}^{2}$/Z/t | *P* value |
| --- | --- | --- | --- | --- |
|  | (n=75) | (n=106) |  |  |
| Age | 63.4±7.7 | 61.6±7.5 | -1.570 | 0.116 |
| Male（%） | 51(68.0) | 84(79.2) | 2.93 | 0.087 |
| Hypertension（%） | 54(72.0) | 50(47.2) | 11.079 | 0.001^**^ |
| Diabetes（%） | 22(29.3) | 26(24.5) | 0.520 | 0.471 |
| Heart disease（%） | 5(6.7) | 14(13.2) | 2.000 | 0.157 |
| History of stroke（%） | 30(40.0) | 24(23.1) | 5.924 | 0.015^*^ |
| Smoking（%） | 34(45.3) | 56(52.8) | 0.987 | 0.32 |
| Drinking（%） | 32(42.7) | 55(51.9) | 1.496 | 0.221 |
| Microbleeds（%） | 18(24.0) | 24(22.6) | 0.045 | 0.831 |
| Years of education | 9(6.0-12.0) | 12(9.0-15.0) | -3.604 | 0.001^**^ |
| Apoe carries E4（%） | 7(9.7) | 10(9.4) | 0.004 | 0.949 |
| HDL cholesterol (mmol/L) | 1.0(0.9-1.2) | 0.9(0.9-1.1) | -1.164 | 0.245 |
| CystatinC(mg/L) | 1.0(0.9-1.1) | 1.0(0.9-1.1) | -0.323 | 0.746 |
| Fasting blood glucose (mmol/L) | 5.4(5.0-7.0) | 5.4(5.1-6.2) | -0.067 | 0.946 |
| Triglycerides (mmol/L) | 1.8(1.3-2.0) | 1.5(1.2-1.9) | -2.065 | 0.039^*^ |
| LDL cholesterol (mmol/L) | 2.9(2.4-3.6) | 2.7(2.3-3.3) | -1.943 | 0.052 |
| VitaminB12(pmol/L) | 248(168.0-336.0) | 281(217.0-421.0) | -1.488 | 0.137 |
| Homocysteine (umol/L) | 11.8(10.3-16.1) | 11.4(9.4-14.7) | -1.323 | 0.186 |
| Folic acid (nmol/L) | 5.6(4-8.3) | 6.5(3.7-8.9) | -0.149 | 0.881 |
| Iron (umol/L) | 14.0±6.1 | 15.4±6.6 | -1.418 | 0.158 |
| Ferritin (ng/ml) | 195.5(152.0-287.8) | 147.9(100.2-242.4) | -1.132 | 0.258 |
| Total iron binding (umol/L) | 47(41.3-51.0) | 48(43.0-54.0) | -0.8 | 0.424 |
| Magnesium (mol/L) | 0.9(0.7-0.87) | 0.8(0.7-0.88) | -0.539 | 0.59 |
| Zinc (umol/L) | 13.4±2.3 | 13.5±2.9 | -0.388 | 0.698 |
| Copper (umol/L) | 15(13.0-16.0) | 14.5(13.1-17.1) | -0.637 | 0.524 |
| Glycosylated hemoglobin (%) | 6(5.6-6.8) | 5.9(5.5-6.7) | -0.087 | 0.931 |
| C-reactive protein (mg/L) | 2.6(1.7-3.5) | 2.9(1.7-3.8) | -0.072 | 0.942 |
| D-dimer (ug/L) | 0.4(0.3-0.7) | 0.46(0.3-0.6) | -0.734 | 0.463 |
| Cholesterol (mmol/L) | 4.6(3.9-5.2) | 4.5(3.6-5.0) | -1.457 | 0.145 |
| WMH（%） |  |  | 10.484 | 0.005^**^ |
| Mild | 18(24.0) | 30(28.3) |  |  |
| Moderate | 21(38.7) | 49(46.2) |  |  |
| Severe | 36(34.8) | 27(25.5) |  |  |
| TOAST classification（%） |  |  | 4.631 | 0.327 |
| Aortic atherosclerotic type | 38(50.7) | 45(42.5) |  |  |
| Cardiogenic embolic type | 5(6.7) | 14(13.2) |  |  |
| Arteriolar occlusion type | 23(30.7) | 40(37.7) |  |  |
| Other causes | 6(8.0) | 5(4.7) |  |  |
| Unexplained type | 3(4.0) | 2(1.9) |  |  |
| Infarction above and below the tentorial（%） |  |  | 0.764 | 0.682 |
| Supratentorial infarction | 57(76.0) | 83(78.3) |  |  |
| Infratentorial infarction | 13(17.3) | 19(17.9) |  |  |
| Supratentorial Infratentorial | 9(6.7) | 4(3.8) |  |  |
| Anterior and posterior circulation infarction（%） |  |  | 1.324 | 0.516 |
| Anterior circulation | 52(69.3) | 65(61.3) |  |  |
| Posterior circulation | 16(21.3) | 30(28.3) |  |  |
| Anterior and Posterior circulation | 7(9.3) | 11(10.4) |  |  |
| MMSE | 24(21.0-26.0) | 28(27.0-29.0) | -7.936 | 0.001^**^ |
| CDR | 0.5(0.5-1.0) | 0.5(0-0.5) | -6.869 | 0.001^**^ |
| ADL | 22(20.0-29.0) | 20(20.0-22.0) | -3.487 | 0.001^**^ |

Abbreviation: HDL, High density lipoprotein. LDL,Low density lipoprotein .WMH, white matter hyperintensities. TOAST, Trial of Org 10172 in Acute Stroke Treatment. MMSE, Mini-mental State Examination. MoCA, Montreal Cognitive Assessment. CDR, Clinical Dementia Rating. ADL, Activity of Daily Living.

**P*＜0.05，***P*＜0.01 indicates that the difference is statistically significant .

**Table S2. Comparison of atherosclerosis characteristics between PSCI and PSNCI groups**

|  | PSCI(n=75) | PSNCI(n=106) | χ²/Z/t | *P* value |
| --- | --- | --- | --- | --- |
| Bifurcation plaque length(mm) | 9(6.7-12.6) | 8.5(6.1-11.5) | -0.159 | 0.874 |
| Bifurcation plaque thick(mm) | 2.1(1.6-2.6) | 1.8(1.7-2.7) | -0.179 | 0.858 |
| Carotid artery bifurcation plaque area（mm^2^） | 20.0(12.5-28.3) | 15.8(9.8-29.7) | -0.027 | 0.978 |
| Carotid plaque Crouse score | 8.2(6.2-11.4) | 5.8(3.2-9.6) | -3.819 | 0.001^**^ |
| Number of plaques | 3(2.0-5.0) | 2(1.0-3.0) | -1.316 | 0.188 |
| Plaque location（%） |  |  | 1.100 | 0.577 |
| No plaques | 10(13.3) | 9(8.5) |  |  |
| Unilateral plaques | 15(20.0) | 22(20.8) |  |  |
| Bilateral plaques | 50(66.7) | 75(70.8) |  |  |
| Left carotid artery IMT | 1.1(0.6-1.7) | 0.7(0.6-1.3) | -1.289 | 0.197 |
| Right carotid artery IMT | 0.8(0.6-1.2) | 0.6(0.5-1.2) | -2.735 | 0.006^**^ |
| Bifurcation plaque location（%） |  |  | -0.851 | 0.396 |
| No plaques | 12(16) | 15(14.2) |  |  |
| Unilateral bifurcated plaques | 25(33.3) | 41(38.7) |  |  |
| Bifurcated plaques on both sides | 38(50.7) | 50(47.2) |  |  |
| Degree of stenosis of the common carotid artery（%） |  |  | 9.388 | 0.002^**^ |
| Mild-moderate stenosis | 50(66.7) | 91(85.8) |  |  |
| Severe stenosis | 25(33.3) | 15(14.2) |  |  |
| RMCA velocity of flow(cm/s) | 95(85.0-116.0) | 103(87.0-152.0) | -1.083 | 0.279 |
| RMCA pulsatility index | 0.9(0.8-1.0) | 0.9(0.8-1.0) | -0.883 | 0.377 |
| LMCA velocity of flow(cm/s) | 101(86.0-156.0) | 104(88.0-153.0) | -0.444 | 0.657 |
| LMCA pulsatility index | 1.0(0.8-1.0) | 0.9(0.9-1.1) | -0.433 | 0.665 |
| RACA velocity of flow(cm/s) | 80.0(69.0-90.0) | 85.0(67.0-106.0) | -1.411 | 0.158 |
| RACA pulsatility index | 0.87(0.8-1.0) | 0.87(0.82-1.0) | -0.665 | 0.506 |
| LACA velocity of flow(cm/s) | 87.0(69.0-115.0) | 86.0(69.0-106.0) | -0.744 | 0.457 |
| LACA pulsatility index | 0.9(0.8-1.0) | 0.9(0.8-1.0) | -0.258 | 0.797 |
| RPCA velocity of flow(cm/s) | 54.0(45.0-70.0) | 54.0(39.0-67.0) | -0.87 | 0.384 |
| RPCA pulsatility index | 0.9(0.8-0.9) | 0.9(0.8-1.0) | -1.333 | 0.183 |
| LPCA velocity of flow(cm/s) | 52.0(45.0-65.0) | 52.0(43.0-65.0) | -0.142 | 0.887 |
| LPCA pulsatility index | 0.9(0.8-1.0) | 0.9(0.8-1.0) | -0.381 | 0.703 |
| RVA velocity of flow(cm/s) | 51.0(40.0-61.0) | 44.0(35.0-62.0) | -0.199 | 0.842 |
| RVA pulsatility index | 0.9(0.9-1.0) | 0.9(0.8-1.0) | -0.016 | 0.31 |
| LVA velocity of flow(cm/s) | 44.0(36.0-56.0) | 44.0(35.0-57.0) | -0.568 | 0.57 |
| LVA pulsatility index | 0.9(0.8-1.0) | 0.9(0.8-1.0) | -0.085 | 0.932 |
| BA velocity of flow(cm/s) | 58.0(48.0-65.0) | 61.0(41.0-69.0) | -0.299 | 0.765 |
| BA pulsatility index | 0.85(0.8-0.9) | 0.9(0.8-1.0) | -1.454 | 0.146 |

Abbreviation: IMT, intima-media thickness. RMCA, Right middle cerebral artery . LMCA, Left middle cerebral artery .RACA, Right anterior cerebral artery . LACA, Left anterior cerebral artery. RPCA, Right posterior cerebral artery . LPCA, Left posterior cerebral artery . RVA, Right vertebral artery. LVA, Left vertebral artery . BA, Basilar artery.

**P*＜0.05，***P*＜0.01 indicate that the differences are statistically significant.

**Table S3. General data were compared between follow-up and nonfollow-ups.**

|  | Nonfollow-up | Follow-ups | *P* value |
| --- | --- | --- | --- |
|  | (n=419) | (n=181) |  |
| Age | 63.07 ± 7.51 | 62.35 ± 7.60 | 0.286 |
| Male（%） | 244 (58.23) | 126 (69.61) | 0.055 |
| Hypertension（%） | 259 (62.41) | 104 (57.46) | 0.110 |
| Diabetes（%） | 133 (31.89) | 48 (26.67) | 0.063 |
| Heart disease（%） | 40 (10.05) | 19 (10.73) | 0.803 |
| History of stroke（%） | 127 (30.60) | 52 (28.73) | 0.085 |
| Smoking（%） | 197 (47.36) | 89 (49.44) | 0.108 |
| Drinking（%） | 169 (41.83) | 86 (48.86) | 0.040* |
| Microbleeds（%） | 153 (50.16) | 42 (40.00) | 0.004* |
| Years of education | 9.00 (6.00, 12.00) | 12.00 (9.00, 12.00) | 0.004* |
| Apoe carries E4（%） | 53 (12.65) | 13 (7.18) | 0.049* |
| HDL cholesterol (mmol/L) | 1.01 (0.89, 1.16) | 0.97 (0.86, 1.17) | 0.217 |
| CystatinC(mg/L) | 0.96 (0.87, 1.10) | 0.94 (0.84, 1.09) | 0.286 |
| Fasting blood glucose (mmol/L) | 5.68 (4.98, 6.95) | 5.51 (5.06, 6.83) | 0.326 |
| Triglycerides (mmol/L) | 1.58 (1.17, 2.17) | 1.48 (1.10, 1.86) | 0.030* |
| LDL cholesterol (mmol/L) | 2.75 (2.24, 3.29) | 2.80 (2.35, 3.41) | 0.180 |
| VitaminB12(pmol/L) | 262.00 (192.00, 349.00) | 275.50 (207.75, 393.75) | 0.061 |
| Homocysteine (umol/L) | 12.94 (11.09, 16.71) | 11.75 (9.77, 15.70) | <0.001** |
| Folic acid (nmol/L) | 6.60 (4.56, 9.60) | 6.30 (4.01, 8.80) | 0.151 |
| Iron (umol/L) | 13.90 (10.60, 17.40) | 15.10 (11.80, 19.35) | 0.014* |
| Ferritin (ng/ml) | 193.50 (118.23, 294.35) | 183.20 (120.80, 261.90) | 0.362 |
| Total iron binding (umol/L) | 46.00 (41.82, 50.00) | 47.00 (43.00, 52.00) | 0.006* |
| Magnesium (mol/L) | 0.85 (0.81, 0.90) | 0.85 (0.80, 0.89) | 0.690 |
| Zinc (umol/L) | 12.20 (11.00, 13.80) | 13.20 (11.60, 15.20) | <0.001** |
| Copper (umol/L) | 15.20 (13.70, 17.00) | 14.90 (13.30, 17.00) | 0.163 |
| Glycosylated hemoglobin (%) | 6.10 (5.70, 7.40) | 6.00 (5.60, 6.90) | 0.161 |
| C-reactive protein (mg/L) | 3.02 (2.70, 3.91) | 2.71 (1.72, 3.46) | <0.001** |
| D-dimer (ug/L) | 0.44 (0.32, 0.64) | 0.43 (0.29, 0.59) | 0.256 |
| Cholesterol (mmol/L) | 4.50 (3.82, 5.19) | 4.50 (3.74, 5.12) | 0.506 |
| MMSE | 26.00 (22.00, 28.00) | 27.00 (24.00, 28.00) | 0.008* |
| CDR | 0.50 (0.00, 1.00) | 0.50 (0.00, 1.00) | 0.288 |
| ADL | 22.00 (20.00, 30.00) | 20.00 (20.00, 24.00) | <0.001** |

Abbreviation: HDL, High density lipoprotein. LDL,Low density lipoprotein . MMSE, Mini-mental State Examination. MoCA, Montreal Cognitive Assessment. CDR, Clinical Dementia Rating. ADL, Activity of Daily Living.

**P*＜0.05，***P*＜0.01 indicates that the difference is statistically significant .

**Table S4.** **IPW-weighted logistic regression with multiple imputation for predictors of PSCI at 6 months.**

| Variable | Odds Ratio (OR) | 95% Confidence Interval | P value |
| --- | --- | --- | --- |
| Crouse score (per unit increase) | 1.14 | 1.03–1.25 | 0.01 |
| Baseline MoCA score | 0.67 | 0.59–0.77 | <0.001 |
| Hypertension | 4.05 | 1.69–9.71 | 0.002 |
| Alcohol consumption | 0.36 | 0.15–0.88 | 0.026 |
| Age | - | NS | >0.05 |
| Diabetes mellitus | - | NS | >0.05 |
| Prior stroke | - | NS | >0.05 |
| Smoking | - | NS | >0.05 |
| Note: IPW = inverse probability weighting; MI = multiple imputation; MoCA = Montreal Cognitive Assessment; PSCI = post-stroke cognitive impairment; NS = not significant. Results adjusted for age, sex, education, and vascular risk factors. | | | |

**
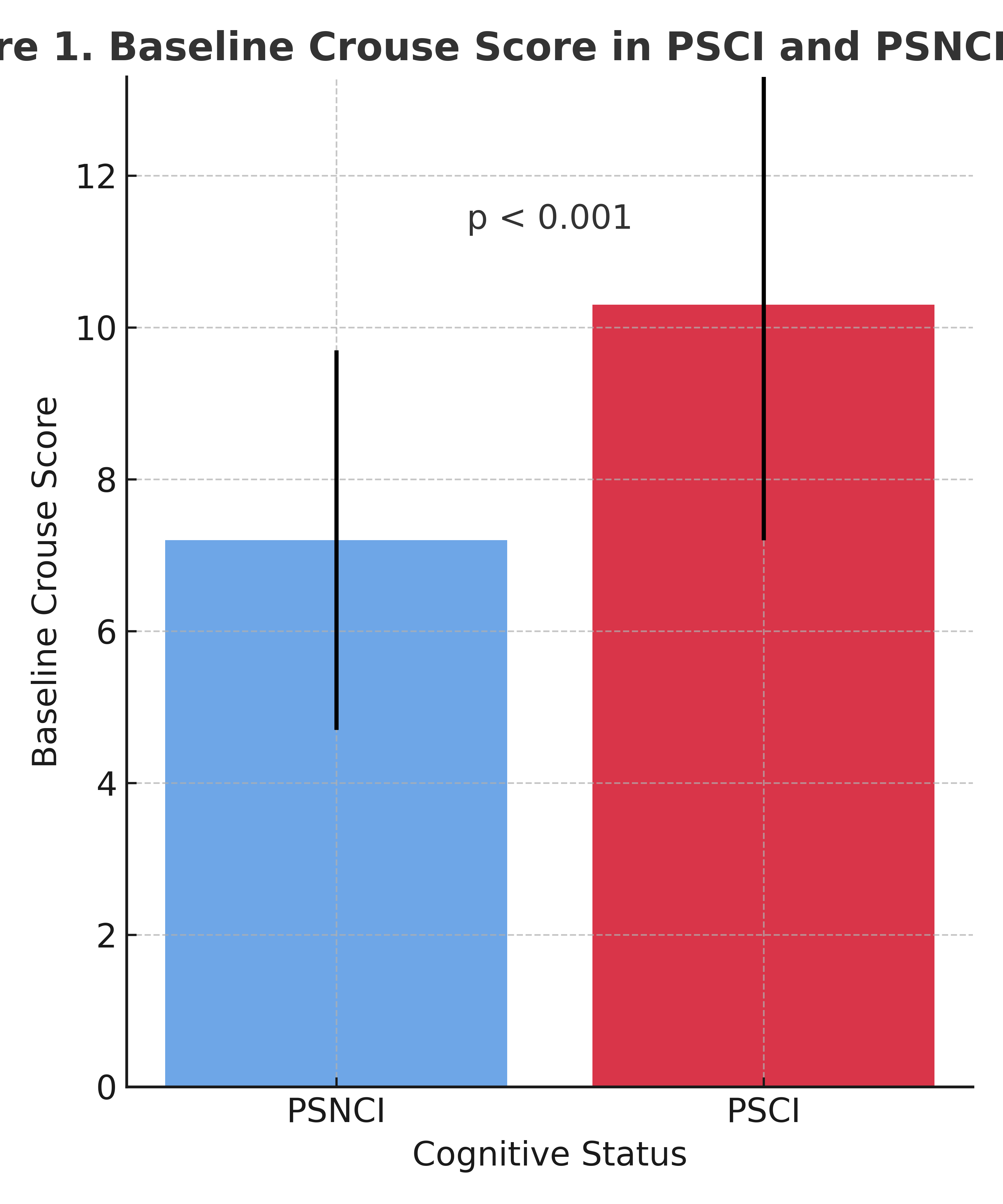
Figure S1. Distribution of Crouse Scores by Post-Stroke Cognitive Impairment Status.**

Abbreviation: Patients with PSCI had significantly higher Crouse scores compared with those without PSCI.

**Figure S2. Proportion of Post-Stroke Cognitive Impairment According to Carotid Stenosis Severity**

**
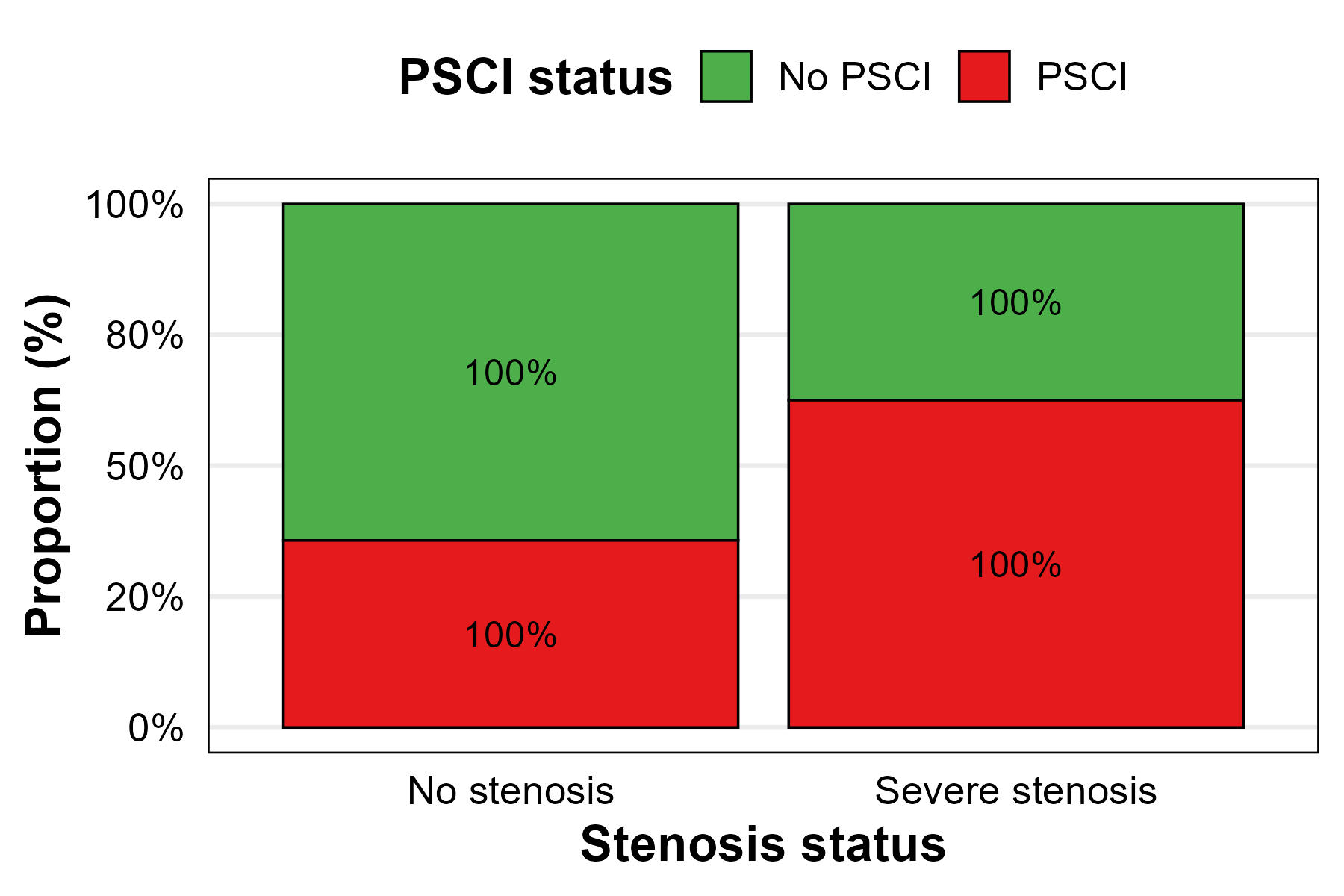
**

Abbreviation: Patients with severe stenosis had a higher proportion of PSCI compared with those without stenosis. Data are shown as percentages within each stenosis category. Green and red bars represent patients without and with PSCI, respectively.
